# The Vaginal Patch Plastron Associated to the Anterior Sacrospinous Ligament Fixation for the Treatment of Advanced Anterior Vaginal Wall Prolapse

**DOI:** 10.3390/jcm11226684

**Published:** 2022-11-11

**Authors:** Alessandro Ferdinando Ruffolo, Benjamin Lambert, Marine Lallemant, Massimo Candiani, Stefano Salvatore, Michel Cosson

**Affiliations:** 1 Department of Gynecology, Jeanne de Flandre University Hospital, 59000 Lille, France; 2 Unit of Gynecology and Obstetrics, IRCCS San Raffaele Hospital, Vita-Salute San Raffaele University, 20132 Milan, Italy; 3 Department of Gynecology, University Hospital of Besançon, 25000 Besançon, France

**Keywords:** pelvic organ prolapse, cystocele, plastron, vaginal patch plastron, anterior sacrospinous ligament fixation, autologous tissue

## Abstract

Background: this study aims to compare the efficacy and safety of vaginal patch plastron (VPP) associated to anterior sacrospinous ligament fixation (SSLF-A) with SSLF-A associated or not to the anterior colporrhaphy (AC) for cystocele treatment. Methods: single-center retrospective study in women with cystocele ≥ III stage submitted to surgery. The primary outcome was to compare objective and subjective cystocele relapse and reoperation rate at follow-up > 6 months. The secondary outcome was to describe peri- and postoperative complications and risk factors for cystocele objective relapse. Results: 75 women were submitted to SSLF-A and 61 women to VPP. VPP objective and subjective relapse (6.5%, 4/61 and 1.1%, 1/61) were lower than SSLF-A (26.7%, 20/75 and 20%, 15/75; *p* = 0.002 and *p* = 0.001, respectively). SSLF-A had a higher reintervention rate, but not significantly (6.6%, 5/75 vs. 0%, 0/61; *p* = 0.06). Previous hysterectomy was a risk factor (HR 4; 1.3–12.1) while VPP was protective factor (HR 0.2; 0.1–0.9) for cystocele anatomical relapse. Postoperative buttock pain was more prevalent in VPP (57.4%, 35/75 vs. 34.7%, 26/61; *p* = 0.01). Conclusions: VPP is effective and safe for advanced cystocele treatment, with lower objective and subjective relapse rates in comparison to isolated SSLF-A or associated with the AC.

## 1. Introduction

Anterior compartment prolapses represent a significant portion of pelvic organ prolapses (POP), with a reported prevalence of 34% in postmenopausal women [1]. A recurrence/reoperation rate of up to 30% after the initial surgery has been described [2], reaching 50% in women submitted to at least 2 POP repair procedures [3].

Because of the recent events related to transvaginal mesh banning in the United States and in Europe, native tissue vaginal repair of POP has returned to the forefront [4].

Historically, in our institute, after the Food and Drug Administration (FDA) warning, sacrospinous ligament fixation performed through an anterior approach (SSLF-A) associated or not to the anterior colporrhaphy (AC) was previously proposed as main vaginal procedure to all patients affected by anterior and/or apical pelvic organ prolapse. However, the observed higher rate of relapse related to this procedure in comparison with transvaginal mesh surgery [5] led to reconsideration of an old vaginal procedure, adopted for the anterior vaginal wall prolapse repair: the vaginal patch plastron (VPP), modified by the association of the SSLF-A for apical suspension. In VPPs, a rectangle of excess vaginal mucosa is used to support the bladder [6]. Traditionally, this rectangle was bilaterally anchored at the arcus tendineus fascia pelvis (ATFP) through three points for each side of the VPP, for a total of six points. The two superior anchors were on the retro-pubic portion of the ATFP, the middle anchors were at midpoint between the ischial spine and the pubic bone, and the inferior anchors were placed 1 to 2 cm above the ischial spine. The VPP, modified by the association of the SSLF-A, substitutes the inferior sutures with a bilateral anterior sacrospinous ligament suspension with the objective of restoring level 1 support [7].

The aim of this study is to evaluate the efficacy and safety of surgical treatment of cystocele by the VPP technique associated with bilateral SSLF-A and to compare this new technique to the SSLF-A isolated or associated with the AC.

## 2. Materials and Methods

This is a single-center retrospective study carried out at the Urogynecological Unit of Jeanne de Flandre Hospital in Lille, France. From March 2019 to September 2022, women affected by anterior vaginal wall prolapse ≥ III stage (Aa or Ba ≥ 1 cm) referred to our center and scheduled for vaginal pelvic surgery were enrolled [8].

Inclusion criteria were: age ≥ 18 years old, symptomatic anterior vaginal wall prolapse ≥III stage (Aa or Ba ≥ 1 cm) according to the pelvic organ prolapse quantification (POP-Q) system, sign of informed consent. Exclusion criteria were: asymptomatic anterior vaginal wall prolapse, follow-up < 6 months, connective tissue diseases, immunodeficiency, auto-immunological diseases, any contraindication to pelvic surgery.

Preoperative evaluation included the record of the medical history, pelvic floor dysfunction (PFD) symptoms (vaginal bulge, urgency urinary incontinence-UUI, stress urinary incontinence-SUI, voiding dysfunction symptoms) and a physical examination, performed by a trained urogynecologist. The terminology used to describe PFD symptoms was in accordance with the standardization document of the International Urogynecological Association and the International Continence Society-IUGA/ICS) [9]. Physical examination was performed in the lithotomy position with maximal Valsalva maneuver, in order to assess POP presence according to the POP-Q system [8].

The two evaluated study procedures were the SSLF-A associated or not with the AC (group 1) and the SSLF-A associated with the VPP (group 2).

The choice of the type of surgical procedure was decided according to the vaginal examination at time of surgery, under regional or general anesthesia, through an intraoperative simulation of postoperative results.

General anesthesia or loco-regional spinal anesthesia were adopted, according to the anesthesiologist’s choice and/or patient’s preference.

Intraoperative data and perioperative complications were recorded.

At the postoperative follow-up visit, medical history, subjective relapse (bulge symptoms) and other PFD symptoms (according to the ICS/IUGA terminology statement), physical examination with evaluation of anatomical outcomes and postoperative complications, in accordance with the Clavien-Dindo classification system, were recorded.

Anatomical anterior vaginal wall prolapse relapse was defined as the presence of a cystocele ≥ II stage (Aa or Ba ≥ −1 cm) according to the POP-Q system. Anatomical apical or posterior compartment relapses were defined as an apical prolapse ≥ II stage (C or D ≥ −1 cm) or a posterior vaginal wall prolapse ≥ II stage (Ap or Bp ≥ −1 cm). Posterior vaginal wall decompensation was defined as the presence of a posterior vaginal wall prolapse ≥ I stage (Ap or Bp ≥ −3 cm), in women who did not present any rectocele or peritoneo/enterocele before the surgical procedure. Subjective relapse was defined as the presence of bulge symptoms, defined as the complaint of a “bulge” or “something coming down” towards or through the vaginal introitus. The woman may have stated she could either feel the bulge by direct palpation or see it aided with a mirror in accordance with the ICS/IUGA terminology statement (9).

At a follow-up longer than 6 months after surgery, women were telephonically questioned about PFD symptoms, POP and incontinence reoperation through the following questions: “Are you still bothered by the feeling of a vaginal ball or something coming down towards or through the vaginal introitus?”, “Have you been operated in another hospital for your prolapse or incontinence after your surgery with us?”, “Do you still have pain in the buttocks or other?”, “Do you have urinary leakage during efforts such as coughing, or sneezing, or during physical activity?”, “Do you have an urge to pee and suddenly have to run to the bathroom? Do you leak urine in this case?”.

The primary outcome of the study was to compare the objective and subjective anterior vaginal wall relapse rate and the POP reoperation rate between the two techniques. The secondary outcome was to identify differences in peri- and postoperative complications and potential risk factors involved in the objective relapse of anterior vaginal wall prolapse after surgery.

All women gave their informed consent in order to be submitted to the surgery allowing us to anonymously use data for research purposes. The study was conducted according to the Declaration of Helsinki.

### 2.1. Procedures

The two procedures can be performed under loco-regional or general anesthesia. We delivered 2 g of Clavulanic Acid—Amoxicillin (intravenous) during the anesthesia’s induction phase. The infiltration is made of a mix of 30 mL lidocaine 1%- and 30-mL normal saline.

#### 2.1.1. Anterior Sacrospinous Ligament Fixation (SSLF-A) and Anterior Colporrhaphy (AC)

The SSLF-A technique has been described and divided into ten surgical steps [10]: exposure, vaginal infiltration, anterior colpotomy, vesico-vaginal dissection, paravesical dissection, sacro-spinous ligament transfixion, vaginal fixation, beginning of the vaginal closure, sacro-spinous ligament fixation and final vaginal closure. Before vaginal closure, AC could be performed through the plication of the Halban fascia.

At the beginning of the procedure, 20 cc of the infiltration mix are injected in the vesicovaginal space and 20 cc in the paravesical space on both sides. After an anterior colpotomy, the paravesical fossa is dissected up to the ischial spine. The SSL running from the ischial spine to the sacral bone is palpated and identified. One suture is placed through the SSL and after the first passage. The same wire can be eventually replaced and a second passage through the SSL is performed. In order to place the suture, we use a suture-capturing device that allows for sutures to be placed easily without eye-control. The same procedure is performed on the other side. Then, the sutures are secured to the cervix or to the top of the vagina if previous total hysterectomy. The sutures used are a non-absorbable, polypropylene monofilament.

The use of vaginal packing is not mandatory at the end of the procedures.

#### 2.1.2. Anterior Sacrospinous Ligament Fixation (SSLF-A) and Vaginal Patch Plastron (VPP)

**Figure 1:**
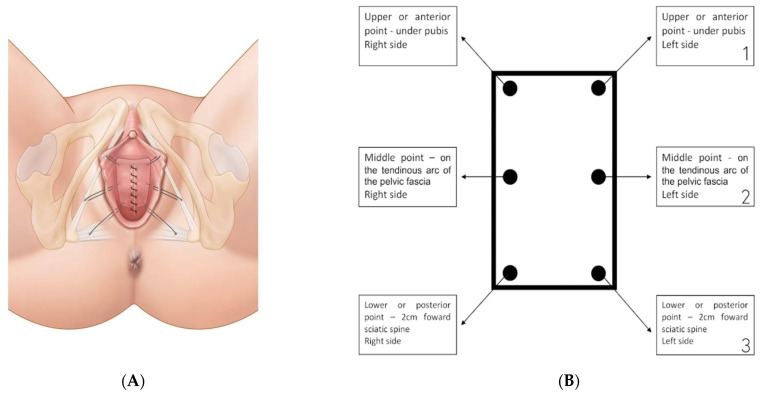
(**A**,**B**) Position of the 6 fixation points of the vaginal patch plastron.

Delimitation of the VPP, a rectangular vaginal strip (about 6–8 cm × 4 cm), on the anterior colpocele. The upper edge of the strip is placed 2 cm from the urethral orifice. Twenty cc of the infiltration mix are injected following the edges of the rectangular vaginal strip and 20 cc in the paravesical space on both sides. The vaginal plastron is then incised and the paravesical spaces are bilaterally dissected up to the ischial spine. The SSL running from the ischial spine to the sacral bone is palpated and identified. One suture is placed through the SSL and after the first passage, the same wire is replaced and a second passage through the SSL is performed with a suture-capturing device that allows sutures to be placed easily without eye-control. Two stitches are placed to the ATFP: a lateral stitch on the tendonal ATFP halfway between the pubis and the sciatic spine and an anterior stitch on the lower edge of pubo-ischial branches. The same procedure is performed on the other side (Figure 1A,B). The sutures used are non-absorbable, polypropylene monofilaments. The vaginal flap is desepidermised in order to prevent the risk of postoperative mucocele, especially in premenopausal women. The VPP will be tied to the SSL and the ATFP by the 3 lateral stitches (upper site for the SSL suture, middle and inferior sites for the 2 arcus tendineus sutures for each side) placed on each side of the VPP. Before the suspension of the VPP, the vaginal closure begins. The VPP is suspended and the vagina is completely sutured.

The use of vaginal packing is not mandatory at the end of the procedures.

### 2.2. Statistical Analysis

The Statistical Package for Social Science (SPSS) version 21.0 was used for data analysis. Continuous variables were reported as mean ± standard deviation (SD) or as median ± interquartile range, as appropriate. The Kolmogorov–Smirnov test was used to analyze the normal distribution of variables (>0.05). The statistical significance of difference in distribution was tested with Pearson chi-square test and the exact Fisher test for categorical variables. For continuous variables, the Mann–Whitney U-test for intergroup differences and the Wilcoxon Rank Sum Test for intragroup differences were adopted as appropriate. The statistical analysis was conducted at a 95% confidence level. Logistic regression was applied to test the association between risk factors for anatomical recurrence. Exploratory univariate analyses were initially applied to all variables, and variables that had a significant association with the adopted scores at univariate analysis (90% confidence level) were eventually included in the multivariate analyses (95% confidence level).

## 3. Results

During the study period, 138 women submitted to anterior vaginal wall prolapse surgery respected the inclusion and exclusion criteria: 76 patients in the SSLF-A with or without AC group (group 1) and 62 patients in the SSLF-A associated with the VPP group (group 2). However, 1 woman in the SSLF-A group (1/76; 1.3%) and 1 woman in the VVP group (1/61; 1.6%) dropped out from the study due to the loss at follow-up and a diagnosis of endometrial cancer 4 months after surgical procedure, respectively. Finally, 136 women, 75 women in group 1 and 61 women in group 2, were included in the study analysis.

General characteristics of the study population are reported in Table 1.

When previous surgical procedures were compared, no statistically significant differences were recorded (Table 2).

At baseline, median POP-Q system score was not significantly different between the two groups for all evaluated points (Aa, Ba, genital hiatus, perineal body, total vaginal length, C, D, Ap and Bp). Baseline POP stages according to the POP-Q system are reported in Table 3. 

Contemporary surgical procedures were similar between groups, as reported in Table 4. Intraoperative data were reported.

Women were discharged the same day of the procedure in 40% (30/75) of patients submitted to SSLF-A and in 31.1% (19/61) of patients submitted to the plastron procedure (*p* = 0.28). When the patients were hospitalized, no differences in the median number of days for discharge were observed (1, IQR 1–2.5 vs. 1, IQR 1–2 days; *p* = 0.76) for group 1 and group 2, respectively.

Last postoperative visit after surgery was at a median of 2.5 months (IQR 2–3.2) in the SSLF-A group and 2.3 months (IQR 2–2.6) in the VPP group (*p* = 0.09).

Anterior, apical and posterior compartment relapses are reported in Table 5.

Objective prolapse recurrence in the VPP group (6.5%, 4/61) was significantly reduced over that in the SSLF-A group (26.7%, 20/75; *p* = 0.002). When the median time of recurrence was evaluated in group 1 (2.5, IQR 2–3 months) in comparison with group 2 (4, IQR 2.5–9 months) no statistically significant differences were highlighted (*p* = 0.47).

A subanalysis was performed in order to evaluate differences in objective outcomes between isolated SSLF-A, SSLF-A with AC and SSLF-A with VPP. When isolated SSLF-A anterior relapse (34.5%; 10/29) was compared with SSLF-A with AC (21.7%; 10/46), no statistically significant differences were observed (*p* = 0.22). However, SSLF-A with VPP recurrence rate resulted significantly inferior both to SSLF-A with AC (*p* = 0.02) and to isolated SSLF-A (*p* = 0.0006).

The incidence of surgical complications assessed with the Clavien–Dindo classification was similar in the 2 patient groups (Table 6), even if the buttock pain resulted significantly more prevalent in the VPP group. However, the mean time of resolution of buttock pain resulted similarly between SSLF-A group and VPP group (17.5 +/− 10.9 days vs. 18.7 +/− 12.3 days; *p* = 0.81), respectively. When obstructive urinary symptoms were postoperatively evidenced, no differences in mean time of intermittent catheterization or indwelling bladder catheter were highlighted between SSLF-A group and VPP group (16.4 +/− 10.5 days vs. 11.2 +/− 7.2; *p* = 0.13).

Women were telephonically investigated at a mean follow up of 14.5 (SD +/− 4.9) months in the SSLF-A group and of 13.5 (SD +/− 5.8) months in the plastron group (*p* = 0.27). A comparison of pre- and postoperative PFD symptoms at follow up is reported in Table 7; bulge symptoms (defined as subjective relapse) in the VPP group (1.1%, 1/61) were significantly reduced compared to the SSLF-A group (20%, 15/75; *p* = 0.001). Even for subjective relapse rate, we performed a subanalysis between isolated SSLF-A, SSLF-A with AC and SSLF-A with VPP. The comparison between isolated SSLF-A (24.1%; 7/29) and SSLF-A with AC (17.4%; 8/46) was not significantly different (*p* = 0.47). Conversely, SSLF-A with VPP resulted in significantly superior results (1.6%; 1/61) both in comparison with isolated SSLF-A (*p* = 0.004) and with SSLF-A with AC (*p* = 0.003). Reoperation rate and type of intervention are reported in Table 5.

Evaluated risk factors for anterior vaginal wall anatomical relapse were reported in Table 8. At univariate analysis both previous hysterectomy and isolated SSLF-A were significantly related, while VPP and the double passage at the sacrospinous ligament were significantly protective; however, only previous hysterectomy was an independent risk factor while the VPP was an independent protective factor for the anterior vaginal wall prolapse objective relapse at multivariate analysis.

## 4. Discussion

In our retrospective study, the vaginal patch plastron technique for the treatment of advanced anterior vaginal wall prolapse (Aa and/or Ba >1 cm) showed a higher objective (93.5% vs. 73.3%; *p* = 0.002) and subjective (98.4% vs. 80%; *p* = 0.001) cure rate in comparison with the anterior sacrospinous ligament fixation, associated or not with anterior colporrhaphy, at a median follow-up between 13.5 and 14.5 months. Moreover, at multivariate analysis, only the VPP resulted as an independent protective factor against cystocele recurrence (hazard ratio 0.2; CI 0.1–0.9).

Definition of success and of failure after anterior POP surgery widely varies among studies [11] with an estimation of failure for the anterior colporrhaphy that largely ranges in the literature from an unbelievable 0% up to a devastating 92% [12]. Therefore, the 6.5% of objective relapse rate observed in the VPP group appears to be low if compared with several studies reported in literature, and in line with the first series of patients submitted to the VPP described in 2001 by Cosson et al. [6], who observed a 6.5% of relapse at a mean of 16.4 months after follow up. Furthermore, all observed anterior vaginal wall prolapse recurrences after VPP were of a low degree (II stage; Aa and/or Ba between −1 and 1 cm), and only the 1.6% of patients complained of bulge symptoms at follow-up.

The association between the VVP technique and the SSLF-A allows the repair of the anterior prolapse at the same time of the apical suspension, leading to a lower objective recurrence rate. Nowadays, it is widely known how the anterior vaginal wall defect coexists with apical compartment defects [7] and that the role of apical suspension is of main importance at the time of anterior vaginal wall prolapse repair in order to reduce the incidence of cystocele relapse [13]. In 2013, Eiber et al. highlighted in women submitted to cystocele repair at a ten-year follow-up that the reintervention rate for anterior prolapse relapse was significantly higher when an isolated anterior surgery was performed (20.2%) in comparison with apical-anterior combined procedures (11.6%; *p* < 0.01) [14].

Concerning the SSLF through an anterior approach, despite the 26.7% objective relapse rate and the 20% subjective relapse rate, our data are in line and sometimes inferior when compared with the current literature, demonstrating to be an effective route to treat anterior prolapse. Indeed, Delacroix et al. described an anterior vaginal wall relapse rate of 37% for the anterior compartment at 10-month follow up after an anterior bilateral approach and a systematic anterior colporrhaphy [15], while Shkarupa et al. reported a higher anterior relapse rate of 45% at 12 months of follow up after a SSLF-A through an apical synthetic sling without performing the AC [16]. At a longer follow-up of 5 years, Jelovsek et al. described a 70% anterior relapse rate, even if they did not differentiate between the anterior and the posterior SSLF approach [17].

It should be considered that our analysis evaluated only advanced cystoceles (III and IV stage according to the POP-Q system), while in the previously mentioned series lower size cystoceles were evaluated too.

Although differences in reoperation rate between the 2 groups did not reach significance, it does not seem a case that all 5 cases of reintervention were reported in the SSLF-A group; we can speculate that the systematic repair of midline and paravaginal defects of the cystocele performed by the VVP may result in a higher solidity of the anatomical results after the vaginal procedure. Indeed, women affected by cystocele relapse in the VPP group were all stage II, with only one patient reporting a postoperative vaginal bulge; all of them were finally surveyed and no further treatments were adopted. Conversely, women in the SSLF-A presented a higher stage of cystocele relapse than the VPP group (8 women presented an anterior prolapse classified stage III and 1 patient stage IV). Fifteen women in the SSLF-A group were symptomatic at follow-up and were treated conservatively by vaginal pessary.

Postoperative buttock pain and urinary retention were observed as the main postoperative complications in both groups. Based on anatomic studies in cadavers, [18] injury to the branches of the sciatic nerve that cross the sacrospinous ligament can lead to postoperative pain or nerve dysfunction, determining the classic triad of nerve entrapment (paresthesias, pain, temporary relief with injection of local anesthetic). Typically, women awaken with severe buttock pain radiating down the posterior leg. In our study, postoperative buttock pain was significantly more prevalent in the VPP group than in the SSLF-A group, probably due to the double passage of the suture at the sacrospinous ligament systematically adopted in group 2. However, this symptom was treated by analgesics and tended to self-resolve between 17 and 19 days after surgery, without differences between the two study groups.

Lower urinary tract complications involving ureteral kinking or injury may also occur during surgery [19]. Our rate of 3.3% of ureteral kinking in the VPP group is consistent with the literature, all treated and solved by ureteral double J.

Postoperative bladder dysfunction is a common complication of gynecologic surgery [20] and ranges from 2 to 43 percent when urogynecological surgery is considered [21]. No differences were observed in our population, and voiding dysfunction generally regressed between 11 and 16 days, after indwelling or intermittent bladder catheterism.

In our opinion, the significant difference of mean operative time (less than 30 min) between the 2 study procedures does not seem to be clinically relevant considering the short term of both surgeries and the mini-invasiveness of the vaginal route. Indeed, one third of women were discharged the same day of surgery while the rest of patients at a median of 1 day.

Previous hysterectomy resulted as the only risk factor for cystocele recurrence at multivariate analysis (hazard ratio 4; CI 1.3–12.1). The role of previous hysterectomy as a risk factor for recurrence after prolapse surgery is controversial. Although a recent meta-analysis of 4 studies did not evidence any relation between previous hysterectomy and POP recurrence after previous pelvic surgery [22], there is evidence of hysterectomy being a risk factor for POP relapse and subsequent prolapse repair [23]. The route of hysterectomy (abdominal, vaginal, laparoscopic or robotic) does not influence recurrence, grade or subsequent treatment of prolapse when the indication for hysterectomy is considered. Only prolapse, as an indication for hysterectomy, increases the risk for recurrence [24].

This study presents several limitations: the evaluation of objective results at short-term follow up, the evaluation of subjective outcomes and of PFD symptoms telephonically and without validated questionnaires and the retrospective design with all intrinsic limits of such design (no randomization, no blinder outcome assessor etc.).

However, we described the largest series of VPP in comparison with a well-standardized technique for POP treatment, with strict inclusion and exclusion criteria in a well-defined population of women. Moreover, a post-hoc power analysis based on the anatomical relapse rate was performed at a 95% of confidence interval and resulted in 89%.

## 5. Conclusions

The vaginal patch plastron associated with the anterior sacrospinous ligament fixation has been proved to be effective and safe for treatment of advanced anterior vaginal wall prolapse. Moreover, the VPP demonstrated to reduce anterior relapse in comparison with the SSLF-A (isolated or in combination with the anterior colporrhaphy) at a follow up longer than one year. Further studies are needed in order to evaluate objective and subjective outcomes at long-term follow-up.

## Figures and Tables

**Table 1:** General characteristics of the study population.

	SSLS-A (n = 75)	VPP (n = 61)	*p* Value
Age, median (IQR), years	70 (65–74)	70 (65.7–73.5)	0.99
BMI, median (IQR), Kg/cm^2^	25.2 (23.3–28.2)	24.6 (22.3–28.9)	93
Vaginal delivery, n (%)	75 (100)	58 (96.7)	0.19
Operative vaginal delivery (forceps), n (%)	18 (24)	10 (16.4)	0.29
Episiotomy, n (%)	34 (44.3)	20 (32.8)	0.16
Cesarean section, n (%)	3 (4)	3 (4.9)	0.79
Smoking habit, n (%)	3 (4)	2 (3.3)	0.82
Menopause, n (%)	74 (98.7)	60 (98.4)	0.88
Sexually active, n (%)	24 (32)	15 (24.6)	0.34

SSLS-A: anterior sacrospinous ligament suspension; VPP: vaginal patch plastron; IQR: interquartile range; BMI: body mass index.

**Table 2:** Previous gynecological and pelvic surgeries of the study population.

	SSLS-A (n = 75)	VPP (n = 61)	*p* Value
Previous hysterectomy, n (%)	16 (21.3)	23 (37.7)	0.06
General previous POP surgery, n (%)	19 (25.3)	19 (31.1)	0.45
Anterior native tissue repair, n (%)	9 (12)	10 (16.4)	0.46
Anterior mesh, n (%)	7 (9.3)	5 (8.2)	0.81
Posterior sacrospinous ligament suspension, n (%)	2 (2.7)	2 (3.3)	0.83
Posterior colporrhaphy, n (%)	3 (4)	5 (8.2)	0.30
Posterior mesh, n (%)	6 (8)	4 (6.5)	0.74
Laparoscopic sacrocolpopexy, n (%)	7 (9.3)	8 (13.1)	0.48
Incontinence surgery, n (%)	13 (17.3)	5 (8.2)	0.11
TVT	6 (8)	3 (4.9)	
TOT	4 (5.4)	2 (3.3)	
Burch	3 (4)	0 (0)	

SSLS-A: anterior sacrospinous ligament suspension; VPP: vaginal patch plastron; POP: pelvic organ prolapse; TVT: tension-free vaginal tape; TOT: trans-obturator tension-free vaginal tape.

**Table 3:** Pelvic organ prolapse stage at baseline according to the POP-Q system.

	SSLS-A (n = 75)	VPP (n = 61)	*p* Value
Anterior vaginal wall prolapse			0.33
- stage III	69 (92)	53 (86.9)	
- stage IV	6 (8)	8 (13.1)	
Apical compartment prolapse			0.06
- stage I	11 (14.7)	6 (10.1)	
- stage II	40 (53.3)	24 (39.3)	
- stage III	11 (14.7)	13 (21.3)	
- stage IV	11 (14.7)	9 (16.4)	
Posterior vaginal wall prolapse			0.85
- stage I	17 (22.7)	15 (24.6)	
- stage II	16 (21.3)	9 (14.7)	
- stage III	2 (2.7)	3 (4.9)	
- stage IV	5 (6.7)	4 (6.5)	

SSLS-A: anterior sacrospinous ligament suspension; VPP: vaginal patch plastron; POP-Q: pelvic organ prolapse-quantification.

**Table 4:** Surgical procedures performed in the study population at time of pelvic surgery and intraoperative data.

	SSLS-A (n = 75)	VPP (n = 61)	*p* Value
Double passage at sacrospinous ligament, n (%)	35 (46.7)	58 (95.1)	<0.0001
Anterior colporrhaphy, n (%)	46 (61.3)	0 (0)	<0.0001
Posterior sacrospinous ligament suspension, n (%)	14 (18.7)	13 (21.3)	0.70
Posterior rectal plication, n (%)	7 (9.3)	6 (9.8)	0.92
Miduretral sling (TVT), n (%)	2 (2.7)	1 (1.6)	0.68
Anesthesia, n (%)			0.49
- General	69 (92)	54 (88.5)	
- Loco-regional	6 (8)	7 (11.5)	
Operative time, mean (SD)	38.5 (±16)	63.3 (+/−27.6)	<0.0001
Surgeon, n (%)			0.81
- Seniors	47 (62.7)	37 (60.6)	
- Trainees	28 (37.3)	24 (39.3)	

SSLS-A: anterior sacrospinous ligament suspension; VPP: vaginal patch plastron; TVT: tension free vaginal tape; SD: standard deviation.

**Table 5:** Anatomical relapse and reoperation rate of the population during the study period.

	SSLS-A (n = 75)	VPP (n = 61)	*p* Value
Anterior vaginal wall objective relapse, n (%)	20 (26.7)	4 (6.5)	0.002
● stage II	11 (14.7)	4 (6.5)	
● stage III	8 (10.7)	0 (0)	
● stage IV	1 (1.3)	0 (0)	
Apical compartment objective relapse, n (%)	5 (6.7)	0 (0)	0.26
● stage II	2 (2.7)	0 (0)	
● stage III	3 (4)	0 (0)	
Posterior vaginal wall objective prolapse relapse °, n (%)	0/14 (0)	1/15 (6.7)	0.99
● stage II	0 (0)	1 (6.7)	
Anatomical decompensation of posterior vaginal wall *, (%)	3/35 (8.6)	3/30 (10)	0.84
● stage I	3 (8.6)	2 (6.7)	
● stage II	0 (0)	1 (3.3)	
Reoperation for anterior vaginal wall prolapse relapse, n (%):	5 (6.6)	0 (0)	0.06
- Laparoscopic sacrocolpopexy	1 (1.3)	0 (0)	
- Robotic sacrocolpopexy	1 (1.3)	0 (0)	
- SSLF-A, AC and SSLF-P	1 (1.3)	0 (0)	
- LeFort partial colpocleisis	2 (2.7)	0 (0)	

° Calculated among women who had posterior vaginal wall prolapse surgery. * Calculated among women who did not have posterior vaginal wall prolapse. SSLS-A: anterior sacrospinous ligament suspension; VPP: vaginal patch plastron; SSLS-P: posterior sacrospinous ligament suspension.

**Table 6:** Clavien–Dindo classification of peri- and postoperative complications.

	SSFA (n = 75)	VPP (n = 61)	Treatment	*p* Value
Grade I				
- Paravaginal hematoma	1 (1.3)	1 (1.6)	Observation	0.88
- Postoperative buttocks pain	26 (34.7)	35 (57.4)	Analgesics	0.01
Grade II				
- Paravaginal abscess	0 (0)	2 (3.3)	Antibiotics	0.19
- Postoperative urinary retention	13 (17.3)	17 (27.9)	Intermittent catheterization/indwelling bladder catheter	0.14
Grade III B				
- Ureteral kinking	0 (0)	2 (3.3)	Double J stent	0.19

SSLS-A: anterior sacrospinous ligament suspension; VPP: vaginal patch plastron.

**Table 7:** Pelvic floor dysfunction symptoms of the study population at follow up.

	SSLS-A (n = 75)	VPP (n = 61)	*p* Value
Bulge symptoms, n (%)	15 (20)	1 (1.6)	0.001
Occult stress urinary incontinence, n (%)	11/50 (22)	11/37 (29.7)	0.41
Persisting stress urinary incontinence, n (%)	7/25 (28)	8/24 (33.3)	0.68
New onset urgency urinary incontinence, n (%)	3/43 (6.9)	8/47 (17)	0.15
Persisting urgency urinary incontinence, n (%)	8/32 (25)	3/14 (21.4)	0.79
New onset voiding urinary symptoms, n (%)	3/51 (5.9)	1/33 (3)	0.64
Persisting voiding urinary symptoms, n (%)	4/24 (16.7)	1/28 (3.6)	0.16

SSLS-A: anterior sacrospinous ligament suspension; VPP: vaginal patch plastron.

**Table 8:** Risk factors for surgical failure and anterior vaginal wall prolapse relapse.

	Univariate Analysis		Multivariate Analysis	
	HR	*p* Value	HR	*p* Value
Age	0.9 (0.9–1.0)	0.34		
BMI	1.1 (0.9–1.2)	0.12		
Previous vaginal delivery	0.9 (0.6–1.2)	0.43		
Cesarean section	1.9 (0.4–8.6)	0.41		
Instrumental vaginal delivery (forceps)	0.7 (0.2–2.3)	0.59		
Episiotomy	1.1 (0.5–2.4)	0.84		
Smoking habits	1.2 (0.1–9.10.9)	0.88		
Previous hysterectomy	2.4 (1.1–5.1)	0.06	4.0 (1.3–12.1)	0.01
Previous POP surgery	2.1 (0.8–5.4)	0.10		
Previous anterior vaginal wall prolapse repair	1.6 (0.5–4.8)	0.43		
Anterior vaginal wall prolapse IV stage	1.3 (0.3–5.1)	0.70		
Central compartment prolapse III stage	2.3 (0.8–6.4)	0.12		
Central compartment prolapse IV stage	1.1 (0.3–3.7)	0.85		
Plastron	0.2 (0.1–0.6)	0.001	0.2 (0.1–0.9)	0.04
Isolated SSLF-A	4.4 (1.7–11.4)	0.003	1.9 (0.6–5.8)	0.23
Double passage at sacrospinous ligament	0.4 (0.1–0.9)	0.04	0.6 (0.2–1.7)	0.31
Anterior colporrhaphy	1.2 (0.5–3.0)	0.67		
Posterior sacrospinous fixation	1.1 (0.4–3.2)	0.89		
Posterior colporrhaphy	0.8 (0.2–4.0)	0.81		

HR: hazard ratio; BMI: body mass index; POP: pelvic organ prolapse; SSLF-A: anterior sacrospinous ligament fixation.

## Data Availability

The data presented in this study are available on request from the corresponding author.
